# Correlation of Serum CysC, IMA, and LP-PLA2 Levels With Type 2 Diabetes Mellitus Patients With Lower Extremity Atherosclerotic Occlusive Disease

**DOI:** 10.3389/fsurg.2022.846470

**Published:** 2022-03-09

**Authors:** Fen Feng, Yong Chen, Gang Wang, Ping Huang, Qiaolin Zhu, Bin Zhou

**Affiliations:** ^1^School of Pharmacy, Shaoyang University, Shaoyang, China; ^2^Department of Oncology and Hematology, The Central Hospital of Shaoyang, Shaoyang, China; ^3^Department of Endocrinology, The Central Hospital of Shaoyang, Shaoyang, China

**Keywords:** type 2 diabetes mellitus, lower extremity atherosclerotic occlusive disease, cystatin C, ischemia modified albumin, lipoprotein phospholipase A2, correlation

## Abstract

**Objective:**

To investigate the serum level of cystatin C (CysC), ischemia-modified albumin (IMA), and lipoprotein-associated phospholipase A2 (LP-PLA2) in patients with type 2 diabetes mellitus (T2DM) and with lower extremity atherosclerotic occlusive disease (LEASOD) and their correlation.

**Methods:**

From March 2017 to December 2019, 110 patients with T2DM with LEASOD, who were treated in our hospital, were selected as the observation group. One hundred ten healthy persons who received medical examination in our hospital during the same period were selected as the control group. Serum CysC, IMA, LP-PLA2, and ankle-brachial index (ABI) were detected in each group. According to the ABI index, the observation group was divided into three subgroups, namely, the mild group (*n* = 45), the moderate group (*n* = 42), and the severe group (*n* = 23). Pearson correlation analysis was used to analyze the relationship between serum CysC, IMA, and LP-PLA2 levels in patients with T2DM with LEASOD and their condition. The receiver operator characteristic (ROC) curve was used to analyze the diagnostic value of serum CysC, IMA, and LP-PLA2 levels in patients with T2DM with LEASOD.

**Results:**

The serum levels of CysC, IMA, and LP-PLA2 in the observation group were higher than those in the control group (*p* < 0.05). The serum levels of CysC, IMA, and LP-PLA2 in the severe and the moderate group were higher than those in the mild group, and the serum levels of CysC, IMA, and LP-PLA2 in the severe group were higher than those in the moderate group (*p* < 0.05). Pearson correlation analysis showed that CysC, IMA, and LP-PLA2 levels were all negatively correlated with ABI (r = −0.802, r = −0.757, r = −0.764, *p* < 0.001). The ROC curve results showed that the area under the curve (AUC) of serum CysC in the diagnosis of T2DM with LEASOD was 0.806, and the best cut-off value was 1.74 mg/L. The AUC of serum IMA for diagnosis of T2DM with LEASOD was 0.772, and the best cut-off value was 92.58 g/L. The AUC of serum LP-PLA2 in the diagnosis of T2DM with LEASOD was 0.781, and the best cut-off value was 544.86 ng/L. The AUC of the three combined diagnoses of T2DM with LEASOD was 0.863.

**Conclusion:**

Serum levels of CysC, IMA, and LP-PLA2 were increased in patients with T2DM with LEASOD. Serum CysC, IMA, and LP-PLA2 are closely related to the severity of the disease. The higher the serum levels of CysC, IMA, and LP-PLA2, the more serious the degree of lower extremity arteriosclerosis occlusion, which can be used as an important serum marker to monitor the severity of T2DM with LEASOD. The combined detection of serum CysC, IMA, and LP-PLA2 has good diagnostic value for patients with T2DM with LEASOD.

## Introduction

Diabetes is a group of metabolic diseases caused by decreased insulin secretion, insensitivity to insulin action of the body, or both, with a chronic increase of blood glucose level and multiple chronic complications as the main clinical features (1, 2). Type 2 diabetes mellitus (T2DM) mainly occurs in middle-aged and older people. In recent years, the incidence of T2DM shows a rising trend. The T2DM can cause microvascular and peripheral vascular lesions, leading to lower extremity atherosclerotic occlusive disease (LEASOD), limb ischemia and intermittent claudication, and even lower extremity ulcer, gangrene, or amputation in the severe cases. LEASOD is one of the most common peripheral arteriosclerosis occlusive diseases in the current society, and it is an important part of systemic arteriosclerosis disease. Its pathological changes are a group of chronic ischemic diseases that cause arterial stenosis or occlusion, such as thickening of the arterial intima, calcification, and secondary thrombosis (3, 4). The incidence of LEASOD is increasing year by year, and 70–80% of patients have no clinical symptoms. Some early manifestations, such as fatigue after exercise and soreness of the lower limbs, are often mistaken by people as a presentation of old age and/or fatigue. Some patients are insensitive to pain due to neuropathy caused by diabetes, and most patients have intermittent claudication, resting pain, ischemic gangrene, and other symptoms when it is difficult to treat, thus, seriously affecting the physical and mental health and quality of life of patients (5, 6).

Cystatin C (CysC) is a cysteine protease inhibitor that is only cleared by a glomerular filtration and almost entirely reabsorbed by the renal tubules. The CysC can regulate the development of inflammation mediated by inflammatory factors and plays an important role in the occurrence and development of atherosclerosis (7, 8). Ischemia modified albumin (IMA) is closely related to coronary artery disease and can be used as an ideal marker of ischemic heart disease (9, 10). Lipoprotein-associated phospholipase A2 (LP-PLA2), a phospholipase, can enter the atherosclerotic plaque under the intima of blood vessels by binding to a low-density lipoprotein, serving as a vascular-specific inflammatory marker (11, 12). The CysC, IMA, and LP-PLA2 are all closely related to vascular lesions and arteriosclerosis. At present, although the technology for diagnosis and treatment of T2DM with LEASOD has become increasingly mature, as a chronic disease requiring continuous medication, it is still very important to seek simple and fast serological indicators to dynamically track the progression of the disease. In this study, the serum levels of CysC, IMA, and LP-PLA2 in patients with T2DM with LEASOD were measured, and the correlation with the severity of the disease was analyzed to make an effective assessment of the degree of lesions in these patients with this index and to provide the basis for clinical diagnosis and treatment.

## Materials and Methods

### Patients

A total of 110 patients with T2DM with LEASOD, who were treated in our hospital from March 2017 to December 2019, were selected as the observation group. The inclusion criteria were as follows: all patients met the diagnostic guidelines for T2DM and lower extremity arteriosclerosis obliterans (13, 14); all patients had a medical history of T2DM with symptoms such as limb pain, intermittent claudication or ischemic ulcer, and gangrene in the lower limbs, and LEASOD was confirmed through color Doppler ultrasound, CT angiography, and other imageological tests to detect the corresponding artery stenosis or occlusion; and all patients should have complete clinical data. The exclusion criteria were as follows: patients who had acute complications of T2DM; patients with hemorrhagic diseases or disorders of the coagulation system; patients with cerebrovascular diseases; patients with severe cardiopulmonary liver and kidney failure; patients with infectious or autoimmune diseases; patients with malignant tumor/s; and patients with alcohol or drug addiction. In the same period, 110 healthy patients, who received physical examination in our hospital, were selected as the control group. There was no significant difference in general data of gender and age between the two groups (*p* > 0.05), indicating that they were comparable as shown in Table 1.

**Table 1:** Comparison of general data between the two groups.

**Group**	**Gender**		**Age (years)**	**BMI (kg/m^**2**^)**
	**Male**	**Female**		
Control group (*n* = 110)	61	49	58.69 ± 3.57	23.38 ± 4.65
Observation group (*n* = 110)	59	51	58.23 ± 3.52	24.19 ± 3.58
t/χ^2^	0.073		0.962	1.448
*P*	0.787		0.337	0.149

### Research Methods

Ten milliliters of fasting blood was collected from the patients in the morning. The blood was allowed to stand at room temperature for 30 min before being centrifuged at 3,500 r/min for 15 min. Afterwards, supernatant was taken and stored in a 2-ml dry and clean serum cold storage tube and stored in −70°C refrigerator for testing. Serum CysC and LP-PLA2 levels were determined by ELISA, and the serum IMA level was determined by the free cobalt colorimetric method.

Relevant test kits were purchased from Today's Chemical Technology (Shanghai) Co., Ltd, which included Ankle brachial index (ABI) measuring instrument and a Doppler blood flow detector EMS-9PB (Shanghai Sanwei Medical Equipment Co., Ltd.).

After a quiet rest for 5 min, the patient was placed in the supine position. The Doppler probe was placed at the brachial artery on the right elbow to obtain the earliest signal. The left upper arm was then measured with the same measuring instrument, and the side with a high value of both arms was taken as the brachial artery systolic blood pressure. The Doppler probe obtained the earliest signal from the right dorsal artery of the foot (or posterior tibial artery) and was then used as the same meter to measure the left foot. The side, with the high value of the left and right feet, was taken as the ankle systolic blood pressure. With the ABI index as the index of the severity of arteriosclerosis obliterans in the lower extremities (ABI = ankle systolic pressure/brachial systolic pressure), lower extremity ischemia could be diagnosed if ABI ≤ 0.90. A lower ABI index indicated that the severity of LEASOD was more serious (15).

According to ABI index, the observation groups were divided into the following three subgroups: mild group (*n* = 45): 0.80 ≤ ABI ≤ 0.90, moderate group (*n* = 42): 0.60 ≤ ABI < 0.80, and severe group (*n* = 23): ABI < 0.60.

### Statistical Methods

All data were processed with SPSS 22.0 statistical software, and GraphPad prism 8 was used to make statistical graphs. The *t-*test was used for pairwise comparison of measurement data between groups. Pearson correlation analysis was used for correlation analysis. The ROC curve was used to analyze the diagnostic value of serum CysC, IMA, and LP-PLA2 levels in patients with T2DM with LEASOD. The difference was considered to be statistically significant if *p* < 0.05.

## Results

### Comparison of Serum CysC, IMA, and LP-PLA2 Levels Between the Two Groups

**Figure 1:**
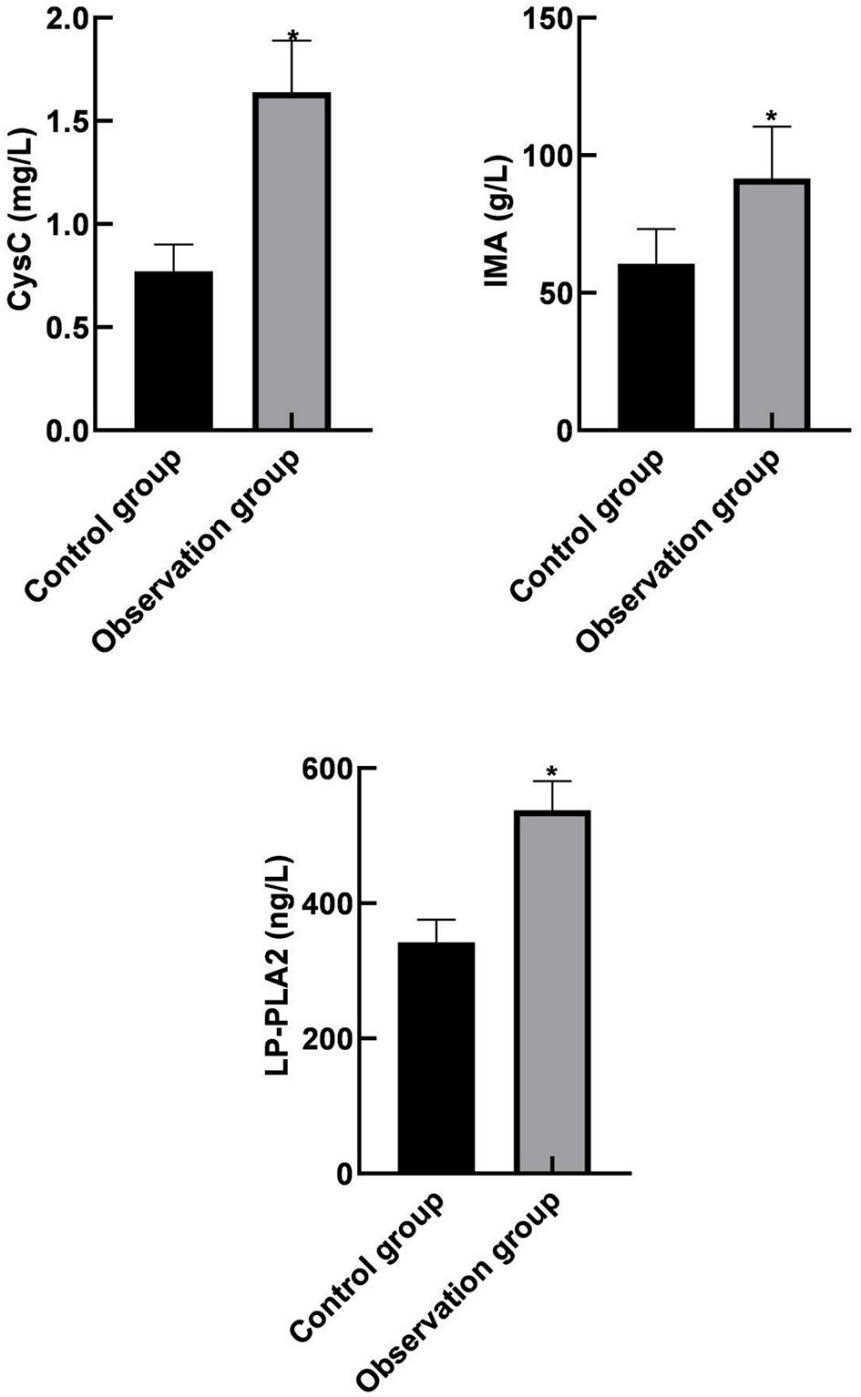
Comparison of serum cystatin C (CysC), ischemia modified albumin (IMA), and lipoprotein phospholipase A2 (LP-PLA2) levels between the two groups. Compared with the control group, *p < 0.05.

Serum CysC, IMA, and LP-PLA2 levels in the observation group were higher than those in the control group (*p* < 0.05) as shown in Figure 1.

### Comparison of Serum CysC, IMA, and LP-PLA2 Levels Among Different Subgroups in the Observation Group

**Figure 2:**
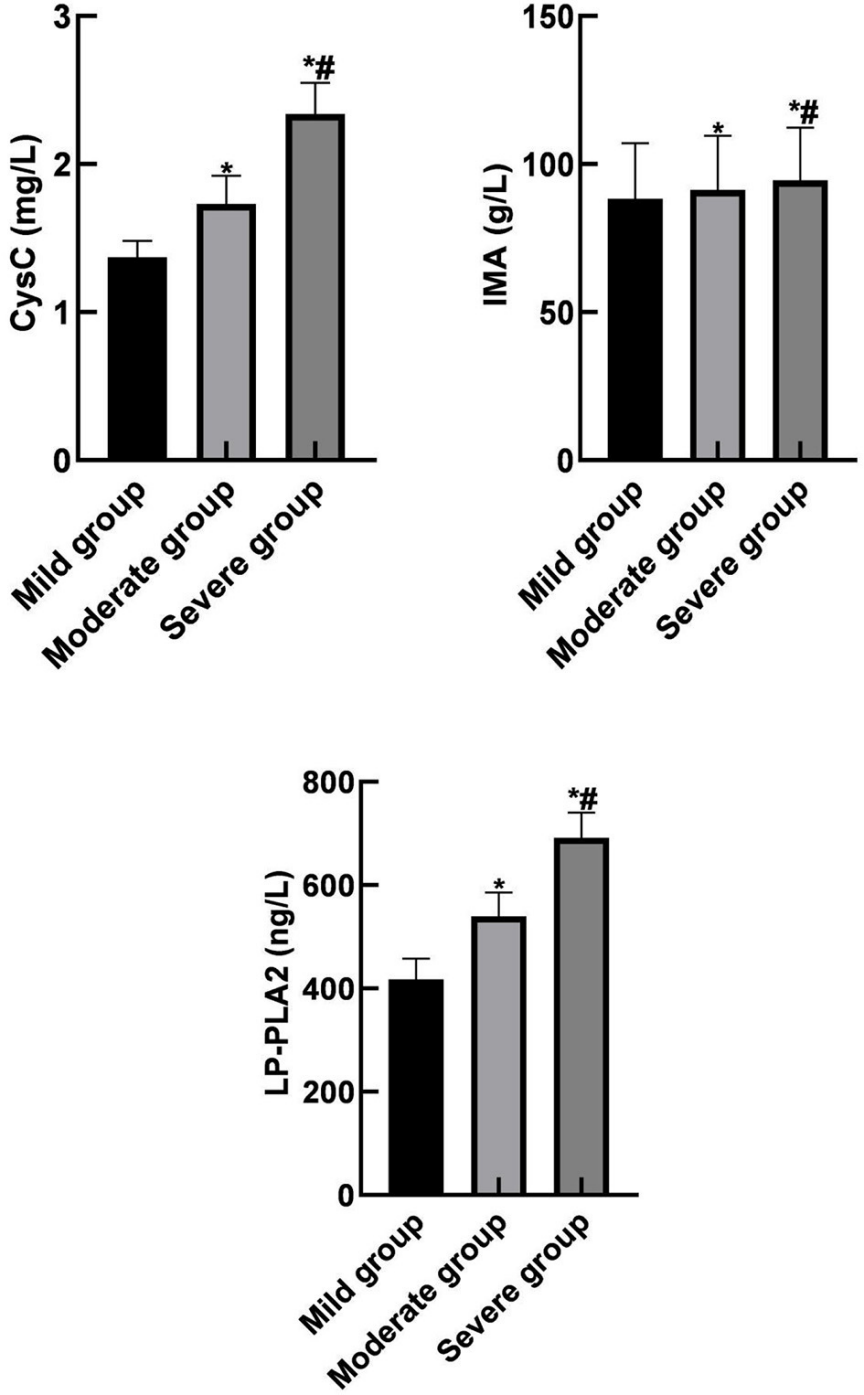
Comparison of serum CysC, IMA, and LP-PLA2 levels among different subgroups in the observation group. Compared with the mild group, *p < 0.05; compared with the moderate group, ^#^*p* < 0.05.

Serum CysC, IMA, and LP-PLA2 levels in the severe and the moderate group were higher than those in the mild group. In addition, serum CysC, IMA, and LP-PLA2 levels in the severe group were higher than those in the moderate group (*p* < 0.05) as shown in Figure 2.

### Correlation Between Serum CysC, IMA, and LP-PLA2 Levels and the Severity of the Disease in the Observation Group

**Figure 3:**
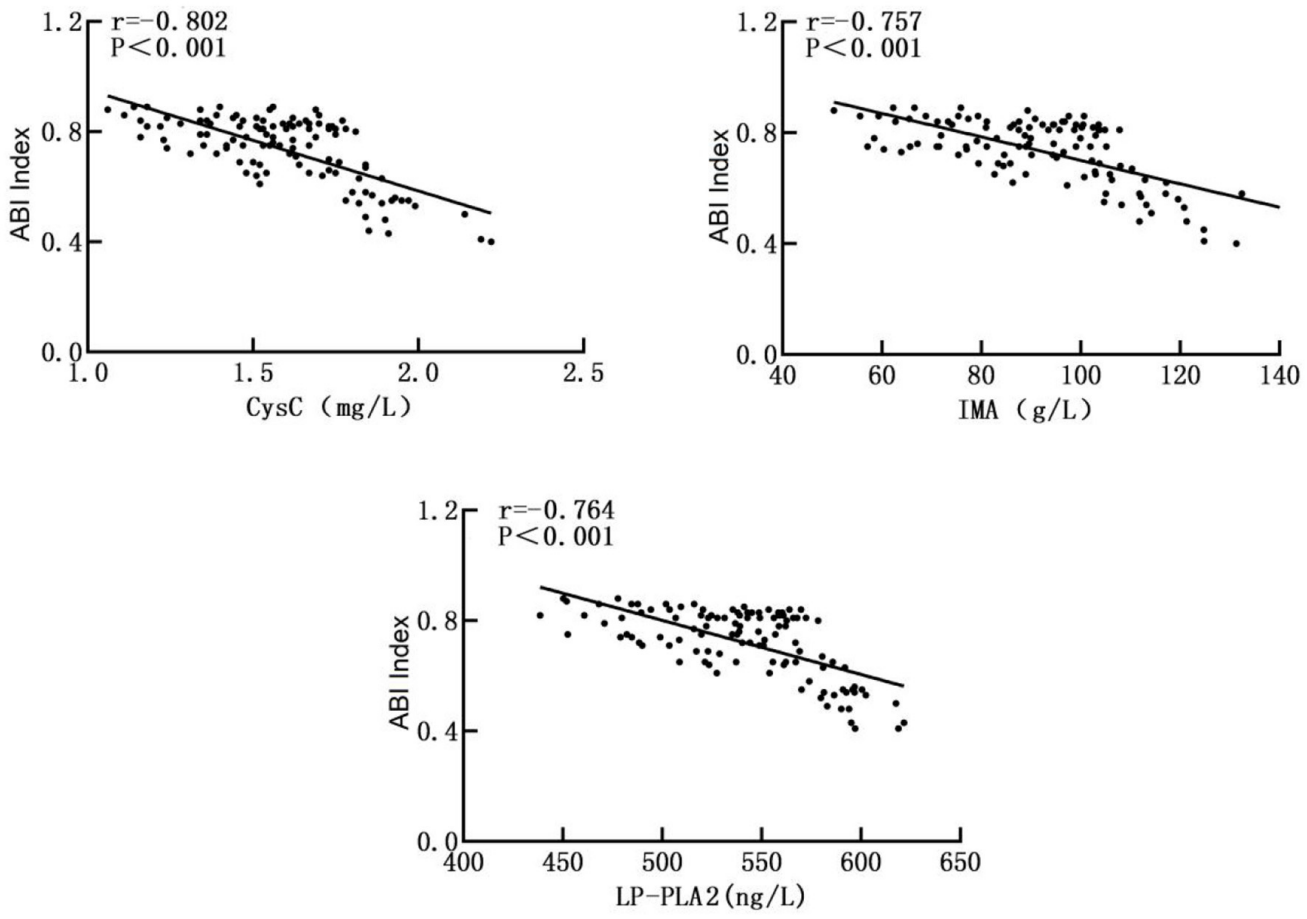
Correlation between serum CysC, IMA, and LP-PLA2 levels and the severity of the disease in the observation group.

Pearson correlation analysis showed that serum CysC, IMA, and LP-PLA2 levels were negatively correlated with the ABI index (r = −0.802, r = −0.757, r = −0.764, *p* < 0.001). Therefore, they were all positively correlated with the severity of the disease as shown in Figure 3.

### Analysis of the Diagnostic Value of Serum CysC, IMA, and LP-PLA2 Levels in Patients

**Figure 4:**
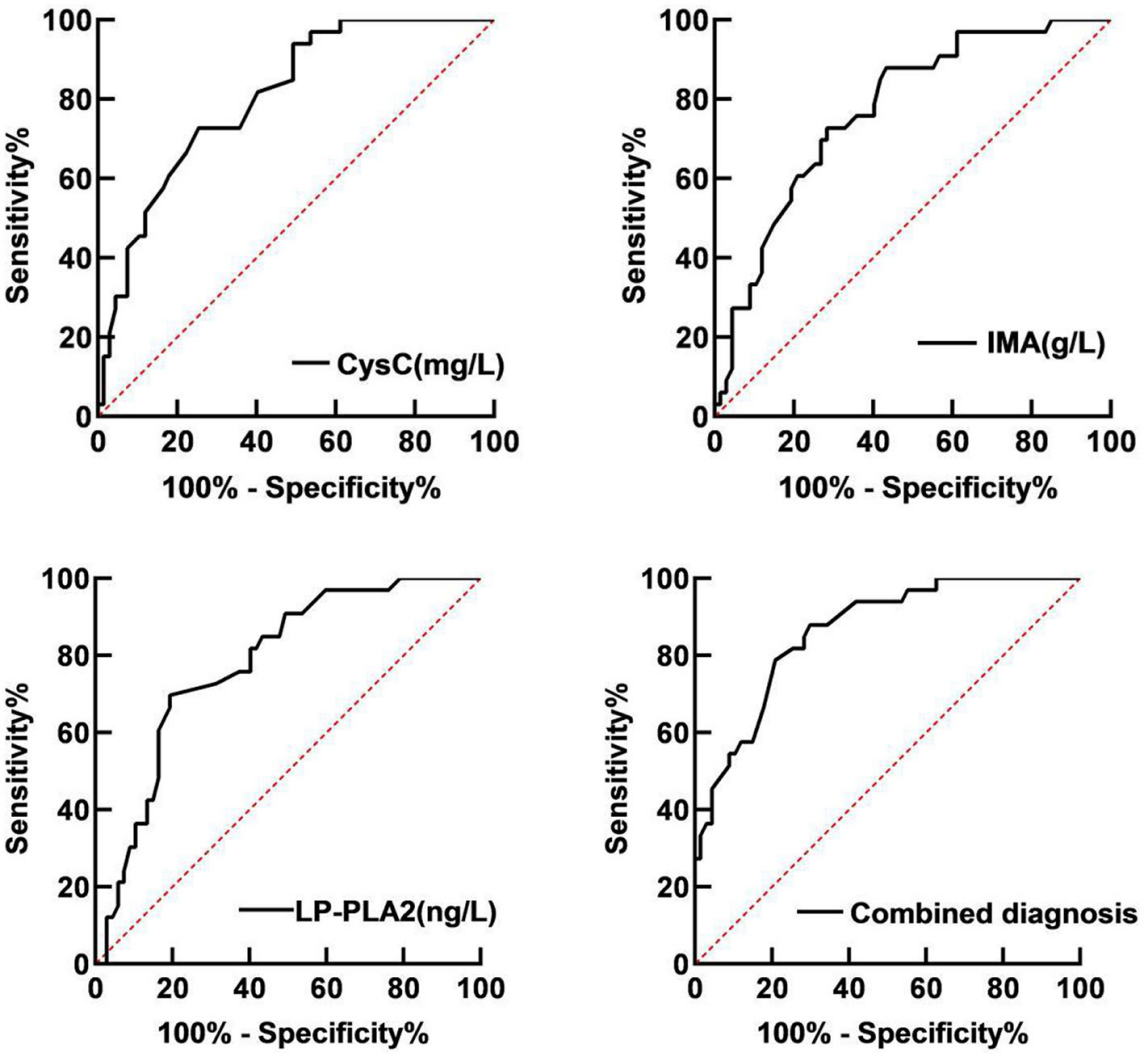
Receiver operator characteristic (ROC) curve of serum CysC, IMA, and LP-PLA2 for the diagnosis of patients with T2DM with LEASOD.

Receiver operator characteristic (ROC) curve analysis showed that the area under the curve (AUC) of serum CysC in the diagnosis of T2DM with LEASOD was 0.806 (95% CI: 0.720–0.891). When the best cut-off value was 1.74 mg/L and the Youden index was 0.446, the sensitivity was 87.9% and the specificity was 66.7%. The AUC of serum IMA in the diagnosis of patients with T2DM with LEASOD was 0.772 (95% CI: 0.678–0.866). When the best cut-off value was 92.58 g/L and the Youden index was 0.503, the sensitivity was 69.7% and the specificity was 80.6%. The AUC of serum LP-PLA2 for the diagnosis of T2DM with LEASOD was 0.781 (95% CI: 0.689–0.872). When the best cut-off value was 544.86 ng/L and the Youden index was 0.473, the sensitivity was 72.7% and the specificity was 74.6%. The AUC of the three combined diagnoses of patients with T2DM with LEASOD was 0.863 (95%CI: 0.791–0.935). When the Youden index was 0.580, the sensitivity was 87.9% and the specificity was 70.1% (Table 2; Figure 4).

**Table 2:** Analysis of the diagnostic value of serum cystatin C (CysC), ischemia modified albumin (IMA), and lipoprotein phospholipase A2 (LP-PLA2) levels in patients.

**Predictive indexes**	**AUC**	**95%CI**	**Youden index**	**Sensitivity (%)**	**Specificity (%)**	**Cut-off value**
CysC	0.806	0.720–0.891	0.446	87.9	66.7	1.74 mg/L
IMA	0.772	0.678–0.866	0.503	69.7	80.6	92.58 g/L
LP-PLA2	0.781	0.689–0.872	0.473	72.7	74.6	544.86 ng/L
Combined predictive	0.863	0.791–0.935	0.580	87.9	70.1	–

## Discussion

In recent years, the prevalence of diabetes has risen sharply and has become one of the important chronic diseases affecting human health with patients with T2DM in the majority (16–18). LEASOD is one of the serious complications of diabetes. It is caused by the influence of a long-term hyperglycemia on the arterial wall, leading to atherosclerosis embolism in the middle layer of the artery, obstruction of lower limb microcirculation, blockage of peripheral nerves, and the formation of diabetic lower limb vascular lesions. It leads to chronic ischemia of and organic lesions of the lower limb. If not treated in time, irreversible damage may eventually be life threatening and may result in amputation in severe cases (19, 20). Therefore, early diagnosis and timely understanding of the condition of the patient is extremely important. The ABI index is characteristically non-invasive, simple, and highly specific. The systolic blood pressure of the ankle in normal people is basically equal to or slightly greater than the systolic blood pressure of the brachial artery. In patients with LEASOD, due to the stenosis or occlusion of the artery of the lower limb, the blood perfusion of the distal artery is reduced, and the arterial pressure of the ankle is decreased, which is roughly proportional to the severity of the disease. Clinically, ABI is commonly used to detect the peripheral artery disease of lower limbs (21, 22). In this study, the severity of LEASOD disease was assessed by ABI index.

Serum crystatin C (CysC), a secretory protein with a small molecular weight, is a cysteine protease inhibitor which is mainly distributed in the extracellular fluid. The highest concentration of CysC can be found in the cerebrospinal fluid and the lowest is in urine (23, 24). Serum CysC can freely pass through the glomerular filtration membrane and degrade after being mostly reabsorbed by the proximal renal tubules. It is not secreted by the renal tubules; hence, the serum concentration is basically determined by glomerular filtration, which is a very ideal endogenous marker reflecting the glomerular filtration rate. In recent years, it was found that CysC is closely connected with cerebrovascular disease, may be involved in many pathophysiological processes of the cardiovascular system, can be involved in inflammation and the extracellular matrix reconstruction process, and is closely related to the development of the occurrence of arterial sclerosis. Some scholars believe that the loss of CysC and the imbalance of proteolytic enzymes and their inhibitors in the vascular wall may be one of the pathogenesis of atherosclerosis (25, 26).

The results of this study showed that serum CysC level in the observation group was significantly higher than that in the control group and that there was a significant negative correlation between CysC level and ABI index. These results suggested that CysC might be involved in the occurrence of T2DM with LEASOD. In addition, the higher the level of CysC, the more serious the LEASOD condition would be, which was positively correlated with the severity of the disease. This is due to how CysC is a low-molecular-weight basic non-glycated protein, the level of which is determined by glomerular filtration rate, which can mediate the inflammatory response and lead to arteriosclerosis through the regulation of inflammatory factors and cytokines. In addition, CysC can inhibit the oxidative low-density lipoprotein-induced apoptosis of the vascular smooth muscle cells to stabilize plaques, thereby causing arteriosclerosis obliteration of the lower limbs. Therefore, CysC can be used as a serum marker for monitoring the severity of LEASOD.

At present, IMA is an ideal marker of ischemia, which has been widely studied mainly in acute coronary syndrome, pulmonary embolism, and cerebrovascular ischemic lesions (27, 28). IMA is an isomer converted into the n-terminus of plasma albumin by exposure to ischemia and oxidative stress, which is caused by changes in the amino terminus sequence of plasma albumin that reduce the ability of metal-binding to the n-terminus of human albumin. In some clinical and experimental studies to date, the elevation of IMA is mainly dependent on oxidative stress after acute ischemia. IMA is elevated during organ ischemic necrosis and generally returns to normal within a few hours after the reperfusion has resumed (29, 30). Therefore, serum IMA levels can be used as important reference indicators when predicting ischemia of atherosclerotic lesions, which is partially consistent with the results of this study. It indicated that IMA was involved in the occurrence of T2DM combined with LEASOD, which was in a significant negative correlation with the ABI index. The higher the level of IMA was, the more serious the condition of LEASOD was, which was in a positive correlation with the severity of the disease. Therefore, IMA can be used as a serum marker for monitoring the severity of LEASOD.

Lipoprotein phospholipase A2 (LP-PLA2) is a new vascular-specific inflammatory marker, also known as plasma platelet activator acetylhydrolase, which is mainly secreted by macrophages and lymphocytes in atherosclerotic plaques and regulated by inflammatory mediators (31, 32). The LP-PLA2 in plasma mainly existed in the form of lipoprotein particle binding, of which about 70–80% is bound to a low-density lipoprotein cholesterol to promote the occurrence of inflammatory reaction, while the remaining 20–30% of LP-PLA2 is bound to a high-density lipoprotein cholesterol or a very low-density lipoprotein cholesterol to play an important role in the anti-oxidation and anti-atherosclerosis effects (33, 34). A prospective study from Sweden found a 4.4% incidence of peripheral artery disease in 5,500 middle-aged men, without a diagnosis of peripheral artery disease and with a mean follow-up of 23.4 years. In addition, it was found that plasma LP-PLA2 activity and quality are risk markers for peripheral vascular disease (35). The results of this study showed that LP-PLA2 was highly expressed in patients with T2DM with LEASOD, and it was significantly and negatively correlated with the ABI index, suggesting that the higher the level of LP-PLA2 was, the more serious the condition of LEASOD was, and it was positively correlated with the severity of the disease. This was due to the fact that LP-PLA2 is a serine esterase that promotes the secretion of inflammatory factors and produces lipid proinflammatory substances, which act on the inner epidermis to dysfunction of the vascular wall, leading to atherosclerosis. Therefore, LP-PLA2 can be used as a serum marker for monitoring the severity of LEASOD.

The ROC curve analysis of this study showed that the AUC of serum CysC, IMA, and LP-PLA2 in patients with T2DM with LEASOD were 0.806, 0.772, and 0.781, respectively, and the AUC of patients with a combined diagnosis of the three was 0.863. It shows that the diagnostic value of the combined diagnosis of serum CysC, IMA, and LP-PLA2 is significantly higher than that of a single diagnosis and has a better diagnostic value.

## Conclusion

The serum levels of CysC, IMA, and LP-PLA2 were increased in patients with T2DM with LEASOD. Serum CysC, IMA, and LP-PLA2 are closely related to the severity of the disease. The higher the serum levels of CysC, IMA, and LP-PLA2, the more serious the degree of lower extremity arteriosclerosis occlusion, which can be used as an important serum marker to monitor the severity of T2DM with LEASOD. The combined detection of serum CysC, IMA, and LP-PLA2 has good diagnostic value for patients with T2DM with LEASOD.

## Data Availability Statement

The original contributions presented in the study are included in the article/supplementary material, further inquiries can be directed to the corresponding author/s.

## Ethics Statement

The studies involving human participants were reviewed and approved by the Ethics Committee of Shaoyang Central Hospital. The patients/participants provided their written informed consent to participate in this study.

## Author Contributions

FF is responsible for the writing of the article. YC is responsible for the design of the study. GW is responsible for the inclusion of cases. PH is responsible for the evaluation of the results. QZ is responsible for data recording and statistics. BZ is the instructor of the entire study. All authors contributed to the article and approved the submitted version.

## Funding

This study was funded by the Hunan Provincial Health Commission Project Fund (202203063365).

## Conflict of Interest

The authors declare that the research was conducted in the absence of any commercial or financial relationships that could be construed as a potential conflict of interest.

## Publisher's Note

All claims expressed in this article are solely those of the authors and do not necessarily represent those of their affiliated organizations, or those of the publisher, the editors and the reviewers. Any product that may be evaluated in this article, or claim that may be made by its manufacturer, is not guaranteed or endorsed by the publisher.
